# The impact of knowledge transfer performance on the artificial intelligence industry innovation network: An empirical study of Chinese firms

**DOI:** 10.1371/journal.pone.0232658

**Published:** 2020-05-18

**Authors:** Guofeng Shi, Zhiyun Ma, Jiao Feng, Fujin Zhu, Xu Bai, Bingxiu Gui

**Affiliations:** 1 School of Economics and Management, Beijing Forestry University, Beijing, China; 2 School of Economics and Management, Ningxia University, Yinchuan, China; 3 School of Management and Economics, Beijing Institute of Technology, Beijing, China; 4 Centre for Artificial Intelligence, School of Computer Science, Faculty of Engineering and IT, University of Technology Sydney, Sydney, Australia; 5 School of Humanities, Tsinghua University, Beijing, China; Institute for Advanced Sustainability Studies, GERMANY

## Abstract

As a core driving force of the most recent round of industrial transformation, artificial intelligence has triggered significant changes in the world economic structure, profoundly changed our life and way of thinking, and achieved an overall leap in social productivity. This paper aims to examine the effect of knowledge transfer performance on the artificial intelligence industry innovation network and the path artificial intelligence enterprises can take to promote sustainable development through knowledge transfer in the above context. First, we construct a theoretical hypothesis and conceptual model of the innovation network knowledge transfer mechanism within the artificial intelligence industry. Then, we collect data from questionnaires distributed to Chinese artificial intelligence enterprises that participate in the innovation network. Moreover, we empirically analyze the impact of innovation network characteristics, organizational distance, knowledge transfer characteristics, and knowledge receiver characteristics on knowledge transfer performance and verify the hypotheses proposed in the conceptual model. The results indicate that innovation network centrality and organizational culture distance have a significant effect on knowledge transfer performance, with influencing factors including network scale, implicit knowledge transfer, receiver’s willingness to receive, and receiver’s capacity to absorb knowledge. For sustainable knowledge transfer performance on promoting Chinese artificial intelligence enterprises innovation, this paper finally delivers valuable insights and suggestions.

## Introduction

Artificial Intelligence (AI) is a strategic technology leading the future. Most developed countries regard the development of AI as a major strategy for enhancing national competitiveness and safeguarding national security. Moreover, several have stimulated policies and strengthened the deployment of core technologies, top talents, and standards, which can enhance nation competitiveness in this most recent round of international technology transformation. As a core driving force of this new round of industrial transformation, AI will further realize the potential of previous scientific and technological revolutions and industrial transformation, creating opportunities for linking production, distribution, exchange, consumption, and other economic activities. The growing demand for a more intelligent life has inspired new technologies, products, and industries, which have led to major changes in our economic structure, production lifestyles, ways of thinking, and overall social productivity [1]. Meanwhile, the effective utilization of AI technology may help achieve sustainable development goals in areas such as health care, climate change, smart city, and poverty eradication.

AI is a complex technology that involves many research areas, such as computer science, philosophy, mathematics, physics, biology, psychology, engineering, linguistics, and logic. Its development requires a wide range of knowledge and relatively high R & D investment. It is believed that we can always achieve innovation goals more efficiently through cooperation. Moreover, the innovation network of the AI industry has already built a platform for R&D cooperation among AI enterprises throughout the world [2]. Since 2015, China has promulgated important national-level strategic plans, such as "Made in China 2025", "Guiding the Opinions of the State Council on Actively Implementing Internet Plus Actions", and the "New Generation AI Development Plan". Local governments have also actively issued policies supporting the development of the AI industry, which has led to an upsurge of AI development in China. At present, the development of Chinese AI is comprised of certain technical and industrial foundations. Specifically, a group of AI companies have gathered from the fields of chips, data, platforms, applications, among others, and have achieved staged results in some areas, developing them toward marketization. For example, AI has been applied in industries such as finance, security, and customer service. In some applications, the accuracy and efficiency of semantic, speech, face, and image recognition technologies have far surpassed manual. At this stage, a number of AI technologies in China, such as speech recognition, visual recognition, machine translation, and Chinese information processing, have reached international levels, forming a relatively complete industrial structure. However, when compared with developed countries, China still exhibits certain gaps in precision parts, the science and technology industry, industrial design, large-scale intelligent systems, and other fields.

Therefore, it is valuable to explore the operation mode of the AI industry innovation network and identify the factors affecting the operation process. There is much existing research on the mechanism behind innovation network operation and knowledge transfer. The AI industry is an emerging industry and an important force in this new era of technological revolution; however, there are still only a few studies on the operation mechanism involved in the AI industry innovation network. In order to explore the operation process of the AI industry innovation network, we empirically studied its operation mechanism of knowledge transfer based on its operation core and channel [3]. Knowledge transfer can occur in different network entities. Specifically, it can be transferred from subjects with high knowledge potential to subjects with low knowledge potential, such as from scientific institutions and universities to enterprises. In this paper, we assume that knowledge transfer occurs between enterprises with high knowledge potential and low knowledge potential. In addition, Chinese AI enterprises have generally fallen behind their foreign counterparts in technical level. Therefore, we postulate foreign AI enterprises as knowledge transfer providers and Chinese local AI enterprises as knowledge receivers.

## Conceptual model and hypothesis

### Conceptual model

Existing research on knowledge transfer models mainly includes research on the knowledge transfer process, the Socialization-Externalization-Combination-Internalization (SECI) knowledge creation model, the five-stage model summarized from the knowledge transfer process, and the knowledge conversion process occurring among the involved organizations. For instance, Hedlund proposed three knowledge transfer steps: coding and internalization, extension and possession, and digestion and diffusion [4]. The SECI knowledge spiral model proposed by Nonaka describes the continuation of tacit knowledge and articulated knowledge through four modes and thus realizing the transfer and creation of knowledge at the various levels of individual, groups organization, and inter-organization [5]. Gilbert proposed five-stage model of knowledge transfer for knowledge transfer behavior between organizations [6]. Szulanski studied a process model that identified the stages of transfer and the factors expected to correlate with difficulties occurring at each [7].

Likewise, Li and Wang highlighted the economics and significance of knowledge transfer in innovation networks from the perspective of clusters and cluster members and analyzed the pathways and influencing factors involved [8,9]. Unconscious knowledge spillovers and imitations of different knowledge transfer models occur frequently in industrial clusters, but the acquisition of tacit knowledge needs to be achieved through long-term interactions among network members [10]. In the process of knowledge transfer in the AI industry innovation network, enterprises are the acting main body [11]. Based on the foregoing analysis, we assumed that excellent foreign AI enterprises are knowledge transfer providers, Chinese intelligence enterprises are knowledge receivers, and that the performance of knowledge transfer in local enterprises depends on both learning source (foreign enterprises) and learning subjects (domestic enterprises) factors as well as the characteristics of knowledge itself. Additionally, it also depends on the different situational factors of the domestic enterprises included in the learning process. These factors not only include friendly interactions that promote organizational knowledge transfer but exclusion factors that hinder knowledge transfer due to organizational cultural distance and geographical distance as well [12]. Therefore, the structural characteristics, organizational distance, knowledge characteristics, and individual organizational factors of the innovation network will affect the performance of knowledge transfer. These factors constituted the independent variables in our conceptual model. In this paper, we also defined the dependent variable as the knowledge transfer performance. Moreover, we investigated the innovation network knowledge transfer performance from the perspective of Chinese AI enterprises via a questionnaire survey.

**Fig 1:**
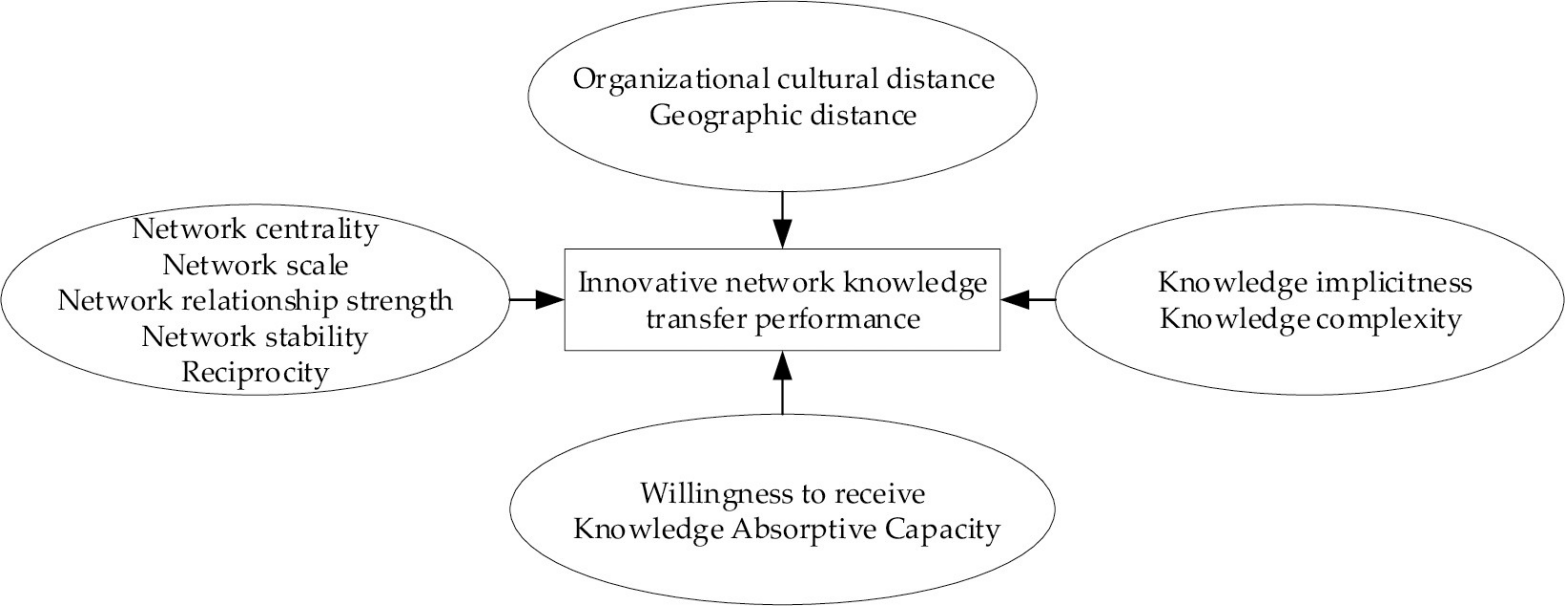
Conceptual model of AI industry innovation network knowledge transfer mechanism.

Building upon research from the existing literature, we integrated the influencing factors of the knowledge transfer between organizations into four domains: innovation network characteristics, organizational distance, characteristics of transferred knowledge, and knowledge receiver factors, each of which consist of several factors. Fig 1 shows the conceptual model proposed in this paper, which we will analyze in the following sections.

### Theoretical hypothesis

We summarize the influencing factors on the knowledge transfer occurring between innovation network subjects from the following perspectives.

#### Innovative network characteristics

Combined with the dimension division of enterprise innovation network characteristics by Chen [13], we divided the characteristics of the AI industry innovation network into two dimensions: the structure dimension and the relationship dimension. The structural dimension mainly refers to the location, scale, and density of network nodes within the network. We selected two indicators of the structural dimension: network centrality and network scale. The relationship dimension is used to express the relationship between the innovation network main body and different network nodes. In this paper, the most representative three indicators were selected for analysis: relationship strength, stability, and reciprocity. Relationship strength reflects the frequency of the connection between the network main body (AI enterprises at home and abroad, universities and scientific institutions, non-governmental organizations (NGOs), financial institutions, and intermediaries), while relationship stability mainly examines the relationship between the AI enterprise and their peers, universities, NGOs, financial institutions, and intermediaries in terms of technology exchange and cooperation. Relationship stability affects the accuracy of knowledge transfer from the network. Reciprocity refers to the degree of exchange information symmetry occurring between subjects in an innovation network. The mutually beneficial network relationship is the symbol of equality between the two partners.

If the AI industry innovation network relationship of Chinese enterprises can influence their organizational learning ability and opportunities, then it will inevitably further affect the enterprise knowledge transfer performance. Therefore, the effective management of the innovation network relationship is the key for Chinese AI enterprises to obtain a large amount of effective information. Moreover, Chinese AI enterprises may improve knowledge transfer performance by improving learning ability.

According to the above review, we propose the following research hypotheses:

**Hypothesis 1 (H1).** The characteristics of the AI industry innovation network are positively related to the knowledge transfer performance of Chinese AI enterprises.

**H1a.** AI industry innovation network centrality is positively related to the knowledge transfer performance of Chinese AI enterprises.

**H1b.** AI industry innovation network scale is positively related to the knowledge transfer performance of Chinese AI enterprises.

**H1c.** AI industry innovation network relationship strength is positively related to the knowledge transfer performance of Chinese AI enterprises.

**H1d.** AI industry innovation network relationship stability is positively related to the knowledge transfer performance of Chinese AI enterprises.

**H1e.** AI industry innovation network reciprocity is positively related to the knowledge transfer performance of Chinese AI enterprises.

#### Organizational distance

We defined organizational distance as the cultural and geographical distance between the network subjects in the innovation network. Since different partners come from diverse contexts, organizational distance refers to the distance generated by organizational structure, organizational skills, institutional traditions, and cultural habits. Specifically, the cultural distance between network subjects in the innovation network refers to differences in cultural traditions and values between the two stakeholders in the knowledge transfer. A similar organizational culture and value system facilitates a smooth exchange between knowledge transfer providers and knowledge recipients; however, if the cultural distance between organizations is too large, this cultural distance may cause differences in the understanding and decoding of knowledge information of different organizations due to differences in language and cultural concepts, thus causing misunderstandings and conflicts in the process of knowledge transfer [14]. Ahammad presented an empirical study on 324 multinational corporations and revealed that cultural distance has a significant negative adjustment effect on the knowledge transfer of transnational parent enterprises [15]. Organizational geographic distance refers to the distance between the geographical locations of cooperative organizations. Simonin studied the influencing factors on knowledge transfer by examining 147 multinational corporations as samples. His findings indicated that, the greater the organizational geographic distance, the worse the knowledge transfer performance among organizations [16]. Pina indicated that the intensity of knowledge transfer depends on the geographical distance between the two regions [17]. Yang indicated that knowledge transfer has spatial interdependence. The diffusion and restraint mechanism of social networks and industrial networks can curb the opportunistic betrayal of the cooperation between organizations and improve the integrity of cooperative relationships [18]. Additionally, Dong found that knowledge exchange and the geographical characteristics of the labor market also play an important role in the innovation and knowledge transfer processes [19]. Meanwhile, Dickens argued that the number of firms established geographically near a university is significantly positively related to the region's intellectual capacity and the university's knowledge output [20]. In summary, geographical distance has an important impact on knowledge transfer.

Based on the above review, we propose the following research hypotheses:

**Hypothesis 2 (H2).** Organizational distance is negatively related to the knowledge transfer performance of Chinese AI enterprises.

**H2a:** The cultural distance between network entities in the innovation network is negatively related to the knowledge transfer performance of Chinese AI enterprises.

**H2b:** The geographical distance between network entities in the innovation network is negatively related to the knowledge transfer performance of Chinese AI enterprises.

#### Knowledge transfer content

Transferred knowledge is the object of knowledge transfer. Its characteristics affect the transfer difficulty of knowledge transfer providers as well as the difficulty of knowledge receivers in receiving the transferred knowledge. Based on the expressiveness of knowledge, we divided knowledge into two subgroups: articulated and tacit knowledge. Besides, knowledge can also be divided into public knowledge and private knowledge based on its existing form within the innovation network environment. Therefore, the characteristics of knowledge include its implicitness, privacy, and complexity [21]. The implicitness of knowledge affects the effects of knowledge transfer by affecting the ability of receiving organizations to understand the knowledge, which makes the transfer process more ultimately complicated. In the innovation network, private knowledge is distributed and shared among the members of the AI innovation network, which is comprised of the stored network rules, procedures, or standardized knowledge. As the knowledge shared by network members, as long as the enterprises join the network, they can obtain it free of charge. In summary, in the innovation network knowledge transfer, we do not consider the privacy of knowledge in the process of knowledge transfer but mainly study the influence of the implicit and complex content of knowledge transfer on the knowledge transfer performance of AI enterprises.

Based on the above review, we propose the following research hypotheses:

**Hypothesis 3 (H3).** The fuzziness of the transferred knowledge in the AI industry is negatively related to the knowledge transfer performance of Chinese AI enterprises.

**H3a.** The implicitness of the transferred knowledge in innovation networks is negatively related to the knowledge transfer performance of Chinese AI enterprises.

**H3b.** The complexity of the transferred knowledge in innovation networks is negatively related to the knowledge transfer performance of Chinese AI enterprises.

#### Subjects of knowledge transfer

The subjects in a knowledge transfer process include both the knowledge transfer provider and the knowledge receiver. On one hand, the influencing factors of the knowledge transfer provider on knowledge transfer performance mainly consist of the transfer intention and the knowledge transfer ability. On the other hand, the factors influencing the knowledge transfer performance of knowledge receivers mainly consist of the willingness to receive knowledge and the ability to absorb knowledge. We assume that, in the process of knowledge transfer, knowledge is mainly transferred from excellent foreign AI enterprises to Chinese AI enterprises. However, the questionnaire given to the foreign enterprises was difficult to issue and retrieve, making the data difficult to obtain. Therefore, we only issued questionnaires to domestic enterprises, assuming that Chinese AI enterprises were the recipients of knowledge. The willingness to receive knowledge and knowledge absorption capacity are the two main impacts that influence knowledge receivers in absorbing the knowledge during the process of knowledge transfer.

The willingness to receive mainly refers to the willingness of the knowledge receiver to receive the knowledge transferred by the knowledge transfer provider. In the process of knowledge transfer, most knowledge receivers can absorb the transferred knowledge more effectively. However, psychologist Surana presented that a proactive attitude can help receivers grasp knowledge more efficiently during the learning knowledge process. Conversely, a negative attitude will hinder the absorption of new knowledge [22]. Therefore, the recipient’s willingness to receive will greatly affect the knowledge transfer performance.

Absorptive capacity refers to the ability of the knowledge receiver to recognize, absorb, and apply the knowledge transferred by the transferor. The absorptive capacity of knowledge recipients is quite important in the process of knowledge transfer [23]. The absorptive capacity of knowledge recipients is mainly related to their comprehension ability and the degree of their knowledge reserve. Zhu indicated that the learning effect of knowledge seekers is limited by their degree of experience. Whether they can effectively absorb the transferred knowledge and apply it to their research field is affected by their existing knowledge stocks [24].

Based on the above review, we propose the following research hypotheses:

Hypothesis 4 (H4).

The knowledge receiver’s own factors are positively related to the knowledge transfer performance of Chinese AI enterprises.

**H4a.** Developers’ willingness to receive knowledge is positively related to the knowledge transfer performance of Chinese AI enterprises.

**H4b.** Developers’ knowledge absorption capacity is positively related to the knowledge transfer performance of Chinese AI enterprises.

## Definitions and measurements

### Measurement of innovative network characteristics

According to the conceptual model introduced in section 2.1, we can see that the AI industry innovation network feature is an explanatory variable. The AI industry innovation network feature measurement items are shown in Table 1.

**Table 1:** Network feature measurement items.

Network feature	Measurement items
Network centrality	The enterprises are dominant in the cooperation network. (WLTZ1)
	In the innovation network, the cooperative R & D process between enterprises can only be accomplished by the participation of the enterprise. (WLTZ2)
	In the innovation network, enterprises can transfer information to other network entities without relying on additional enterprises. (WLTZ3)
Network scale	Number of enterprises in the innovation network. (WLTZ4)
	Number of universities and scientific institutions in the innovation network. (WLTZ5)
	Number of NGOs in the innovation network. (WLTZ6)
	Number of financial institutions in the innovation network. (WLTZ7)
	Number of intermediaries, such as consulting enterprises, in the innovation network. (WLTZ8)
Relationship strength	Frequency of communication between an enterprise and other companies in the innovation network. (WLTZ9)
	Frequency of communication between an enterprise, universities, and scientific institutions in the innovation network. (WLTZ10)
	Frequency of communication between an enterprise and NGOs in the innovation network. (WLTZ11)
	Frequency of communication between an enterprise and financial institutions in the innovation network. (WLTZ12)
	Frequency of communication between an enterprise and intermediary in the innovation network. (WLTZ13)
Relationship stability	Duration of cooperation between enterprise and other companies in the innovation network. (WLTZ14)
	Length of cooperation between an enterprise, universities, and scientific institutions in the innovation network. (WLTZ15)
	Length of cooperation between an enterprise and NGOs in the innovation network. (WLTZ16)
	Length of cooperation between an enterprise and financial institutions in the innovation network. (WLTZ17)
	Length of cooperation between an enterprise and intermediary in the innovation network. (WLTZ18)
Reciprocity	Whether Chinese AI enterprises and enterprises in the innovation network exchange their own confidential information with each other. (WLTZ19)
	Whether Chinese AI enterprises and enterprises in the innovation network fulfill their commitments to each other. (WLTZ20)
	Even when the opportunity arises, Chinese AI enterprises and their partners in the innovation network will not take advantage of each other. (WLTZ21)
	Whether Chinese AI enterprises and enterprises in innovation network trust each other. (WLTZ22)

### Measurement of organizational distance

We measured organizational distance in two dimensions: cultural distance and geographic distance. Cultural distance is defined as the difference between AI enterprises in terms of the cultural backgrounds of the different countries. According to a study by Simonin, cultural distance is measured by three aspects [25]. Likewise, in the measurement of geographical distance, we referred to the study by Cummings, proposing three items, as shown in Table 2 [26].

**Table 2:** Organizational distance measurement items.

Organizational distance	Measurement items
Cultural distance	There are often cross-cultural conflicts or misunderstandings when knowledge transfer occurs among different subjects of an innovation network. (WLJL1)
	Language barrier is the main obstacle to communication with other enterprises. (WLJL2)
	Perceiving that the cultures of other enterprise’s countries are very different from their own. (WLJL3)
Geographic distance	The geographical distance between the enterprises and their partner enterprises in the innovation network is far. (WLJL4)
	The geographical distance between the enterprises, universities, and scientific institutions in the innovation network is far. (WLJL5)
	The geographical distance between the enterprises and the technology intermediary agencies, non-governmental organizations, and other institutions in the innovation network is far. (WLJL6)

### Measurement of knowledge transfer content

Knowledge implicitness refers to the extent to which the knowledge transferred by the knowledge transfer provider cannot be easily transmitted through text, language, or graphic symbols during the operation of the innovation network, and it is difficult to transfer or share to the knowledge receiver. Knowledge complexity refers to the characteristic that, during the operation of the innovation network, the knowledge transferred by the knowledge transfer provider cannot be simply absorbed and digested by the receiver and applied to the R & D process of the enterprise. In this paper, we combined the measurement terms of knowledge implicitness and knowledge complexity presented by Shu [27] and Ma [28] and selected three measurement items, as shown in Table 3.

**Table 3:** Knowledge transfer content measurement items.

Knowledge transfer content	Measurement items
Knowledge implicitness	Knowledge cannot be clearly expressed or explained in a written form, such as language, diagrams, and text, during the process of knowledge transfer among innovative enterprises in the innovation network. (ZSTX1)
	There are more parts of the transfer that are difficult to describe in the process of knowledge transfer among innovative enterprises in the innovation network. (ZSTX2)
	There is more empirical and technical content in the process of knowledge transfer among innovative enterprises in the innovation network. (ZSTX3)
Knowledge complexity	There are many different fields involved in the process of knowledge transfer of innovative enterprises in the innovation network. (ZSTX4)
	Knowledge is the combination of multiple interdependent technologies, procedures, and resources in the process of knowledge transfer among innovative enterprises in the innovation network. (ZSTX5)
	Enterprises require individuals from different departments to learn together in the process of knowledge transfer among innovative enterprises in the innovation network. (ZSTX6)
	Enterprises must learn in a frequent and informal way in the process of knowledge transfer among innovative enterprises in the innovation network. (ZSTX7)

### Measurement of knowledge transfer subjects

The willingness to receive is defined as the degree of willingness of the knowledge receiver to receive the transfer knowledge. We noticed that few studies have focused on the willingness to receive. In this paper, we adopted the terms proposed by Cummings [26] and Szulansk [29] in their relevant research to measure the level of willingness to receive. Moreover, absorptive capacity is defined as the ability of the innovation network subjects to identify, absorb, transform, and apply the transferred knowledge to achieve considerable benefits. We drew upon the relevant measurement clauses of the innovative network subject knowledge receivers by Shu [27] and selected measurement items, as shown in Table 4.

**Table 4:** Knowledge transfer subject measurement items.

Subjects of knowledge transfer	Variable contents
Willingness to receive knowledge	Enterprises regard organizational learning as a key factor in gaining competitive advantage. (ZSJS1)
	Enterprises regard organizational learning process as a long-term investment. (ZSJS2)
	Enterprises regard the organizational learning process as key to their development. (ZSJS3)
Knowledge absorptive capacity	Enterprises have a strong ability to integrate and receive knowledge from outside. (ZSJS4)
	Enterprises are introducing external knowledge at a fast pace. (ZSJS5)
	Enterprises clearly know which external knowledge is helpful. (ZSJS6)

### Measurement of knowledge transfer performance

Knowledge transfer performance is a comprehensive evaluation of the effect of knowledge transfer. In current studies, most scholars regard knowledge transfer as a dependent variable, studying the ways in which knowledge transfer is carried out among transfer subjects and evaluating the results under certain influencing factors.

Some studies have proposed using knowledge transfer costs to measure knowledge transfer performance [30]. Previous research has mainly explored knowledge transfer performance from two perspectives: economic and technical. In terms of the economic indicators, the literature has found that the economic growth rates [31], knowledge transfer costs [32] and market shares [33] of enterprises are positively related to knowledge transfer performance. In terms of the technical indicators, early research has demonstrated that technical and innovation capabilities would facilitate enterprise knowledge transfer performance [34]. Additionally, other studies have also stressed the importance of the knowledge receiver's satisfaction with the knowledge transfer performance [35].

According to our proposed conceptual model, the dependent variable proposed in this paper is the performance of enterprise knowledge transfer in the AI industry innovation network. Drawing on existing research, we adopted Wang's study results on knowledge transfer performance, which measures it using four factors: improving work efficiency, improving the internal innovation success rate, improving market competitiveness, and investing a small amount of human and financial resources [36]. The specific measurement scale is shown in Table 5.

**Table 5:** Knowledge transfer performance measurement items.

Dependent variable	Variable content
Knowledge transfer performance	By acquiring transferred knowledge, the human and financial resources invested in R & D have been reduced. (ZYJX1)
	By acquiring transferred knowledge, enterprises have increased their market competitiveness. (ZYJX2)
	By acquiring transferred knowledge, enterprises have increased their internal innovation success rate. (ZYJX3)
	By acquiring transferred knowledge, enterprises have increased their R & D efficiency. (ZYJX4)

The data in this paper was mainly obtained from a questionnaire surveys collected from Chinese AI enterprises. We first defined the AI companies participating in the innovation network. The definition criteria were based on the introduction on each company's official website and includes international AI technology research and development talents, Sino-foreign joint venture AI enterprises, and foreign-funded AI enterprises. In order to obtain a sufficient sample of effective questionnaires, we defined companies that meet any of the above conditions as AI companies integrated into the innovation network. The selection of this questionnaire was mainly aimed at AI companies participating in technology R&D personnel or middle and senior managers of the enterprise, to ensure the questionnaire participants’ comprehensive understanding of each company's participation in the AI industry innovation network.

The measurement items in this paper were closely centered around the research hypothesis of this study to ensure the content of the questionnaire was consistent with the survey items. At the same time, the design of the questionnaire items account for clarity, conciseness, and logic. Based on the maturity scale in the field of innovation networks, we drew on existing measurement items at home and abroad, and selects those that are widely used, highly recognized, and highly relevant to the purpose of this study. The rest of the questionnaire was structured as follows: The first part is the instructions, which explains the background, purpose, and procedure of the survey. The second part corresponds to basic information about the respondent and company, such as ownership type, enterprise scale, development stage, etc. The third part is the main part of the questionnaire. Additionally, based on the study by Chen [13] and Wang [36], and considering the characteristics of the innovation network, we designed 47 survey questions based on four factors: innovation network characteristics, organizational distance, knowledge characteristics, and knowledge receivers. The questionnaire adopted the Likert 5-scale method, with 1, the lowest score, denoting “extremely disagree” and 5, the highest score, denoting “extremely agree”. Part of the items set the answer standard according to the research questions, and marked in the questionnaire. The descriptive analysis of the data sample is as follows. The proportion of companies in the startup stage was 2.75%; the proportion of companies in the investment stage was 11.95%; the proportion of companies in the growth stage was 69.79%; the proportion of companies in the mature stage was 14.64%; and the proportion of companies in the decline stage was 0.86%.

Additionally, to avoid the ethical issues of this research, the survey was conducted with the respondents' full knowledge and consent. Specifically, on the one hand, the ethical review of the research content was completed by Ningxia university. The project leader reviewed the research in person and gave oral approval. On the other hand, after the questionnaire was sent to the AI enterprise, the personnel department only sent it to the respondents after the ethical review. Meanwhile, at the end of the questionnaire, we set a conspicuous note: If you think this questionnaire violates the ethical issues, you have the right to refuse to complete it.

We used questionnaires and stepwise regression for analysis. The basic concept behind a stepwise regression is to introduce variables one-by-one into the model. After each explanatory variable is introduced, an F-test is performed, and the selected explanatory variables are then individually subjected to a t-test. When it is no longer significant, delete it. This ensures that only significant variables are included in the regression equation before each new variable is introduced. This is an iterative process until no significant explanatory variables are selected into the regression equation and no insignificant explanatory variables are removed from the regression equation in order to ensure that the final set of explanatory variables is optimal.

## Model test and regression analysis

### Factor analysis

In this section, we first tested the validity of the scale (see Table 6). As mentioned above, the content validity of the scale was guaranteed. As a result, we mainly tested the structural validity of the scale. The criterion for the factor analysis was that the KMO statistic had to reach 0.6 or above, and the Bartlett sphericity test needed to meet the significance level of 0.05. In this respect, our study used the statistical software SPSS 19.0 to calculate the KMO statistics and perform the Bartlett sphericity tests. The calculation results met the factor analysis criteria. According to the results of the scale validity test in Table 6, the measured values of the KMO statistics for the four variables and the eleven sub-variables were significantly greater than 0.7. The values of the Bartlett sphericity test were all 0.000, which also met the requirements, indicating that factor analysis can be carried out for the variable correlation matrix selected in this paper. We also analyzed the nominal variables by utilizing Eigenvalues, Cumulative interpretation variation, Factor load, Cronbach’s α and other mensuration as follows.

**Table 6:** Validity test scale results.

Variables		KMO	Significance of the Bartlett test
Innovative network characteristics	Network centrality	0.845	0.000
	Network scale	0.869	0.000
	Relationship strength	0.842	0.000
	Relationship stability	0.758	0.000
	Reciprocity	0.762	0.000
Organizational distance	Cultural distance	0.895	0.000
	Geographic distance	0.854	0.000
Knowledge characteristics	Knowledge implicitness	0.835	0.000
	Knowledge complexity	0.878	0.000
Knowledge receiver characteristics	Willingness to receive	0.841	0.000
	Knowledge absorptive capacity	0.822	0.000

#### Innovation network characteristics factor analysis and reliability test

The factor analysis of the innovation network characteristics structure scale showed that the internal consistency of the scale was strong (see Table 7). The correlation level being between 0.4 and 0.6 indicated that the correlation was at a medium-strong, and the internal consistency of the scale was good. After the revision of this study, the total correlation level of the different items was greater than 0.5, indicating that the internal consistency of the scale was indeed good.

**Table 7:** Factor analysis and reliability test scale results of the innovation network characteristics.

Nominal variables	Operation variables	Factor analysis				Reliability test		
		Deleted item	Eigenvalues	Cumulative interpretation variation /%	Factor load	Cronbach’s α	α of item deleted	Total correlation of the revised item
Network centrality (X1)	WLTZ1	0	3.021	25.661	0.811	0.792	0.701	0.692
	WLTZ2				0.856		0.725	0.755
	WLTZ3				0.844		0.703	0.523
Network scale (X2)	WLTZ4	1	2.152	37.993	0.774	0.787	0.615	0.564
	WLTZ5				0.722		0.608	0.587
	WLTZ6				0.685		0.712	0.522
	WLTZ8				0.611		0.711	0.574
Relationship strength (X3)	WLTZ9	1	2.024	48.511	0.725	0.732	0.638	0.582
	WLTZ10				0.702		0.720	0.538
	WLTZ11				0.672		0.651	0.551
	WLTZ13				0.655		0.645	0.625
Relationship stability (X4)	WLTZ14	1	2.025	61.388	0.755	0.711	0.646	0.557
	WLTZ15				0.735		0.642	0.613
	WLTZ16				0.708		0.625	0.500
	WLTZ18				0.712		0.685	0.662
Reciprocity (X5)	WLTZ19	0	2.011	73.005	0.736	0.745	0.609	0.501
	WLTZ20				0.781		0.728	0.598
	WLTZ21				0.722		0.697	0.601

#### Organizational distance factor analysis and reliability test

We also conducted factor analysis on six items related to the organizational distance factor (see Table 8). The cumulative degree of interpretation of the feature scale reached 51.204%, indicating that the two factors of organizational distance could explain at least 51.204% of the variation in the predictor variable.

**Table 8:** Factor analysis and reliability test scale results regarding organizational distance.

Nominal variables	Operation variables	Factor analysis				Reliability test		
		Deleted item	Eigenvalues	Cumulative interpretation variation /%	Factor load	Cronbach’s α	α of item deleted	Total correlation of the revised item
Knowledge culture distance (X6)	WLJL1	0	3.438	37.558	0.695	0.852	0.842	0.667
	WLJL2				0.753		0.823	0.741
	WLJL3				0.745		0.818	0.599
Geographic distance (X7)	WLJL4	0	1.225	51.204	0.811	0.722	0.523	0.521
	WLJL5				0.825		0.571	0.563
	WLJL6				0.772		0.604	0.505

#### Transferred knowledge characteristics factor analysis and reliability test

We performed factor analysis on seven items related to the transferred knowledge characteristics (see Table 9). Relevant tests showed that the indicators had certain reliability and credibility.

**Table 9:** Factor analysis and reliability test scale results regarding transferred knowledge.

Nominal variables	Operation variables	Factor analysis				Reliability test		
		Deleted item	Eigenvalues	Cumulative interpretation variation /%	Factor load	Cronbach’s α	α of item deleted	Total correlation of the revised item
Knowledge implicitness (X8)	ZSTX1	0	3.856	35.281	0.723	0.802	0.874	0.612
	ZSTX2				0.645		0.854	0.548
	ZSTX3				0.767		0.846	0.623
Knowledge complexity (X9)	ZSTX4	1	3.742	67.152	0.787	0.864	0.838	0.654
	ZSTX5				0.724		0.851	0.625
	ZSTX6				0.762		0.846	0.657

#### Knowledge receiver factor analysis and reliability test

We performed factor analysis on six items related to the two variables of the knowledge receiver factor (see Table 10). The items in these are not deleted.

**Table 10:** Factor analysis and reliability test scale results regarding the knowledge receiver.

Nominal variables	Operation variables	Factor analysis				Reliability test		
		Deleted item	Eigenvalues	Cumulative interpretation variation /%	Factor load	Cronbach’s α	α of item deleted	Total correlation of the revised item
Willingness to receive knowledge (X10)	ZSJS1	0	2.982	35.642	0.801	0.825	0.803	0.576
	ZSJS2				0.748		0.776	0.645
	ZSJS3				0.845		0.766	0.752
Knowledge absorptive capacity (X11)	ZSJS4	0	3.956	66.747	0.564	0.854	0.845	0.563
	ZSJS5				0.742		0.748	0.656
	ZSJS6				0.693		0.856	0.525

#### Knowledge transfer performance factor analysis and reliability test

We performed factor analysis on four items related to the knowledge transfer performance factor (see Table 11). One item of these items was deleted.

**Table 11:** Factor analysis and reliability test scale results of the knowledge transfer performance.

Nominal variables	Operation variables	Factor analysis				Reliability test		
		Deleted item	Eigenvalues	Cumulative interpretation variation /%	Factor load	Cronbach’s α	α of item deleted	Total correlation of the revised item
Knowledge transfer performance(Y)	ZYJX1	1	2.905	59.642	0.785	0.825	0.758	0.725
	ZYJX2				0.855		0.771	0.669
	ZYJX4				0.625		0.813	0.586

### Classified regression analysis

#### Classified regression analysis of the AI industry innovation network characteristics and knowledge transfer performance

The classified regression analysis results showed that network centrality and network scale had a significant effect on knowledge transfer performance, while relationship strength, stability, and reciprocity were negatively related to knowledge transfer performance and failed to pass the significance test (see Table 12).

**Table 12:** Classified regression analysis results of network characteristics and organizational knowledge transfer performance.

Variable	Coefficient	t	Sig.	Collinear statistic	
				Tolerance	VIF
Constant	0.253				
network centrality (X1)	0.228	3.568	0.000	0.715	1.386
Network scale (X2)	0.256	6.077	0.000	0.766	1.552
Relationship strength (X3)	-0.065	0.854	0.245	0.865	1.182
Relationship stability (X4)	-0.011	7.225	0.102	0.689	1.322
Reciprocity (X5)	-0.022	0.287	0.152	0.748	1.224
*R*	*R*^2^	Adjusted *R*^2^		Durbin-Watson	
0.892	0.796	0.725		1.761	

#### Classified regression analysis on the organizational distance between the network subjects and knowledge transfer performance in AI industry innovation network

Table 13 lists the classified regression analysis results regarding organizational distance. The results show that the organizational culture distance (X6) is a significant effect factor on knowledge transfer performance. However, the effect factor of geographical distance (X7), by contrast, was not significant and did not pass the significance test.

**Table 13:** Classified regression analysis results for organizational distance and knowledge transfer performance.

Variable	Coefficient	t	Sig.	Collinear statistic	
				Tolerance	VIF
Constant	0.129				
Culture distance (X6)	-0.329	-3.288	0.000	0.725	1.325
Geographic distance (X7)	-0.022	-6.287	0.385	0.739	1.524
*R*	*R*^2^	Adjusted *R*^2^		Durbin-Watson	
0.822	0.676	0.625		1.852	

#### Classified regression analysis on transferred knowledge characteristics and knowledge transfer performance in AI industry innovation network

Table 14 shows that knowledge implicitness (X8) is significantly related to knowledge transfer performance. Additionally, the knowledge complexity (X9) did not pass the significance test. Thus, it was negatively related to knowledge transfer performance.

**Table 14:** Classified regression analysis results of knowledge characteristics and knowledge transfer performance.

Variable	Coefficient	t	Sig.	Collinear statistic	
				Tolerance	VIF
Constant	0.112				
knowledge implicitness (X8)	-0.221	-6.218	0.000	0.855	1.695
Knowledge complexity (X9)	-0.019	-7.287	0.226	0.869	1.112
*R*	*R*^2^	Adjusted *R*^2^		Durbin-Watson	
0.732	0.536	0.511		1.912	

#### Classified regression analysis of knowledge receivers and knowledge transfer performance in AI industry innovation network

Table 15 presents the classified regression analysis results for the knowledge receivers. The results indicate that both willingness to receive (X10) and absorptive capacity (X11) are significantly related to knowledge transfer performance.

**Table 15:** Classified regression analysis results of knowledge receivers and knowledge transfer performance.

Variable	Coefficient	t	Sig.	Collinear statistic	
				Tolerance	VIF
Constant	0.547				
Willingness to receive (X10)	0.215	6.218	0.000	0.735	1.365
Absorptive capacity (X11)	0.458	2.287	0.000	0.729	1.612
*R*	*R*^2^	Adjusted *R*^2^		Durbin-Watson	
0.852	0.726	0.651		1.522	

In the proposed conceptual model, we also divided the 11 explanatory variables into four categories: innovation network characteristics, organizational distance, characteristics of transferred knowledge, and knowledge receiver factors. Furthermore, through the classified regression analysis of the various variables, we can discuss the effect of the explanatory variables in each category on the dependent variables. The explanatory variables within each category were complete, and there were no missing key variables in the four classified regressions. Here, we summarize the classified regression analysis results, as shown in Table 16. Six of the 11 hypotheses were supported by the existing data (with a significance level of 1%), and the other five hypotheses were not.

**Table 16:** Classified regression analysis results.

Hypothesis	Contents	Validation Results
H1	The characteristics of the AI industry innovation network are positively related to the knowledge transfer performance of Chinese AI enterprises.	Partly Pass
H1a	AI industry innovation network centrality is positively related to the knowledge transfer performance of Chinese AI enterprises.	Pass
H1b	AI industry innovation network scale is positively related to the knowledge transfer performance of Chinese AI enterprises.	Pass
H1c	AI industry innovation network relationship strength is positively related to the knowledge transfer performance of Chinese AI enterprises.	Fail to Pass
H1d	AI industry innovation network relationship stability is positively related to the knowledge transfer performance of Chinese AI enterprises.	Fail to Pass
H1e	The AI industry innovation network reciprocity is positively related to the knowledge transfer performance of Chinese AI enterprises.	Fail to Pass
H2	Organizational distance is negatively related to the knowledge transfer performance of Chinese AI enterprises.	Partly Pass
H2a	The cultural distance between network entities in the innovation network is negatively related to the knowledge transfer performance of Chinese AI enterprises.	Pass
H2b	The geographical distance between network entities in the innovation network is negatively related to the knowledge transfer performance of Chinese AI enterprises.	Fail to Pass
H3	The fuzziness of the transferred knowledge in the AI industry is negatively related to the knowledge transfer performance of Chinese AI enterprises.	Partly Pass
H3a	The implicitness of the transferred knowledge in innovation networks is negatively related to the knowledge transfer performance of Chinese AI enterprises.	Pass
H3b	The complexity of the transferred knowledge in innovation networks is negatively related to the knowledge transfer performance of Chinese AI enterprises.	Fail to Pass
H4	The knowledge receiver’s own factors are positively related to the knowledge transfer performance of Chinese AI enterprises.	Pass
H4a	Developers’ willingness to receive knowledge is positively related to the knowledge transfer performance of Chinese AI enterprises.	Pass
H4b	Developers’ knowledge absorption capacity is positively related to the knowledge transfer performance of Chinese AI enterprises.	Pass

### Stepwise regression analysis

In practice, the explanatory variable of one factor may produce an increase or decrease effect in a multiple interactive factor regression. Accordingly, we performed a stepwise regression analysis of the four groups of variables in this section (see Table 17).

**Table 17:** Stepwise regression analysis results of the knowledge transfer performance impact factors.

Model	Coefficient	Sig.	Collinear		F Value	Adjusted *R*^2^
			Tolerance	VIF		
1 Constant	0.253				26.54 (0.000)	0.796
Network centrality	0.228	0.000	0.715	1.386		
Network scale	0.256	0.000	0.766	1.552		
Network relationship strength	0.065	0.245	0.865	1.182		
Network stability	0.011	0.102	0.689	1.322		
Reciprocity	0.022	0.152	0.748	1.224		
2 Constant	0.153				23.42 (0.000)	0.802
Network centrality	0.201	0.001	0.966	1.022		
Network scale	0.223	0.001	0.975	1.228		
Network relationship strength	0.052	0.263	0.752	1.415		
Network stability	0.121	0.324	0.763	1.455		
Reciprocity	0.012	0.156	0.659	1.568		
Organizational cultural distance	-0.308	0.000	0.825	1.208		
Geographic distance	-0.001	0.359	0.869	1.568		
3 Constant	0.103				18.374 (0.000)	0.755
Network centrality	0.199	0.002	0.945	1.256		
Network scale	0.205	0.101	0.926	1.289		
Network relationship strength	0.043	0.274	0.576	1.698		
Network stability	0.118	0.228	0.522	1.755		
Reciprocity	0.006	0.173	0.573	1.956		
Organizational cultural distance	-0.229	0.000	0.788	1.836		
Geographic distance	-0.000	0.459	0.754	1.255		
Knowledge implicitness	-0.105	0.036	0.569	1.785		
Knowledge complexity	-0.009	0.326	0.2580	1.963		
4 Constant	0.093				16.577 (0.000)	0.655
Network centrality	0.187	0.002	0.954	1.263		
Network scale	0.199	0.051	0.856	1.299		
Network relationship strength	0.037	0.325	0.355	2.568		
Network stability	0.105	0.278	0.762	2.056		
Reciprocity	0.001	0.245	0.552	1.256		
Organizational cultural distance	-0.213	0.000	0.855	1.854		
Geographic distance	-0.000	0.669	0.256	1.165		
Knowledge implicitness	-0.098	0.049	0.564	4.265		
Knowledge complexity	-0.001	0.306	0.235	3.256		
Willingness to receive	0.201	0.076	0.295	2.091		
Absorptive capacity	0.325	0.073	0.566	1.645		

As the stepwise regression results listed in Table 18 indicate, of the 11 hypotheses, only two hypotheses were validated by the existing data at a 1% significance level. This was quite different from the results of the classified regression analysis.

**Table 18:** Stepwise regression analysis results.

Hypothesis	Contents	Validation results
H1	The characteristics of the AI industry innovation network are positively related to the knowledge transfer performance of Chinese AI enterprises.	Partly Pass
H1a	AI industry innovation network centrality is positively related to the knowledge transfer performance of Chinese AI enterprises.	Pass
H1b	AI industry innovation network scale is positively related to the knowledge transfer performance of Chinese AI enterprises.	Fail to Pass
H1c	AI industry innovation network relationship strength is positively related to the knowledge transfer performance of Chinese AI enterprises.	Fail to Pass
H1d	AI industry innovation network relationship stability is positively related to the knowledge transfer performance of Chinese AI enterprises.	Fail to Pass
H1e	AI industry innovation network reciprocity is positively related to the knowledge transfer performance of Chinese AI enterprises.	Fail to Pass
H2	Organizational distance is negatively related to the knowledge transfer performance of Chinese AI enterprises.	Partly Pass
H2a	The cultural distance between network entities in the innovation network is negatively related to the knowledge transfer performance of Chinese AI enterprises.	Pass
H2b	The geographical distance between network entities in the innovation network is negatively related to the knowledge transfer performance of Chinese AI enterprises.	Fail to Pass
H3	The fuzziness of the transferred knowledge in the AI industry is negatively related to the knowledge transfer performance of Chinese AI enterprises.	Fail to Pass
H3a	The implicitness of the transferred knowledge in innovation networks is negatively related to the knowledge transfer performance of Chinese AI enterprises.	Fail to Pass
H3b	The complexity of the transferred knowledge in innovation networks is negatively related to the knowledge transfer performance of Chinese AI enterprises.	Fail to Pass
H4	The knowledge receiver’s own factors are positively related to the knowledge transfer performance of Chinese AI enterprises.	Fail to Pass
H4a	Developers’ willingness to receive knowledge is positively related to the knowledge transfer performance of Chinese AI enterprises.	Fail to Pass
H4b	Developers’ knowledge absorption capacity is positively related to the knowledge transfer performance of Chinese AI enterprises.	Fail to Pass

**Fig 2:**
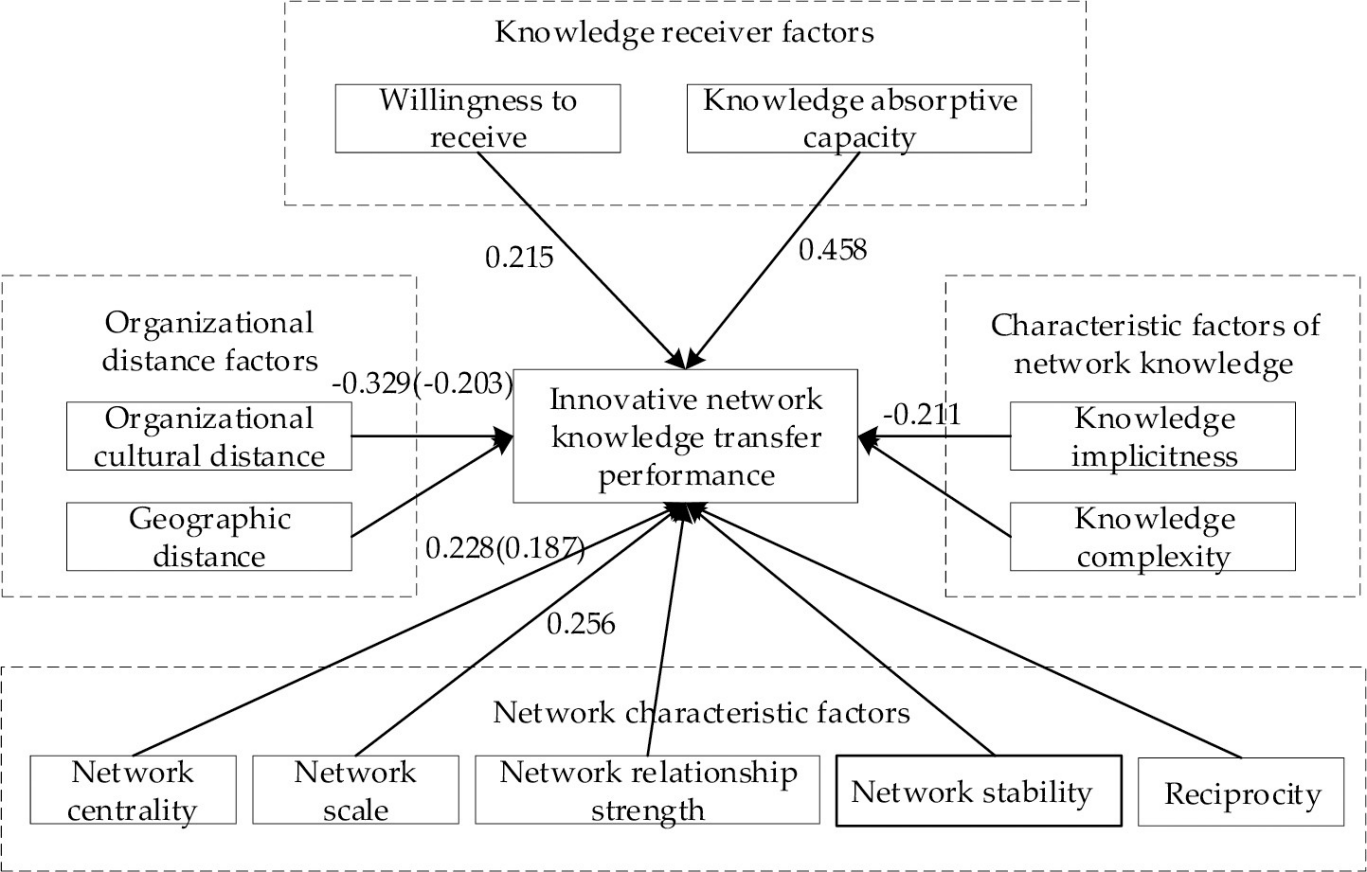
Results of classified regression and stepwise regression analysis.

In order to clearly present the effect factors and direction of the knowledge transfer performance in the AI innovation network, we illustrate the above test results in Fig 2. The independent variable (with the number indicated on the arrow) was tested based on the significance of the classified regression. The number represents the regression coefficient, in which two independent variables, network centrality and organizational culture distance, pass the significance test of the classified regression. The regression coefficient is shown in brackets.

## Discussion

### Discussion of the regression results of the innovation network characteristics as independent variables

1. The relationship between the network centrality and knowledge transfer performance in the AI innovation network. As the classified regression analysis results show, the regression coefficient between the centrality characteristics of the enterprise and the knowledge transfer performance was 0.228 (P < 0.000), and the regression coefficient between the centrality of the enterprise and the knowledge transfer performance in the stepwise regression results (four factors simultaneously as explanatory variables) was 0.187 (P < 0.000), which indicates that the centrality of the network enterprise had a significant and positive effect on the knowledge transfer performance. The greater the enterprise's dominance in the network and the more important role it plays in the network’s information transmission, more new knowledge it absorbs during the knowledge transfer process and the better knowledge transfer performance it demonstrates. In the AI innovation network, enterprises are important carriers of knowledge. Therefore, integration into the innovation network and taking on a certain status are the main ways Chinese AI enterprises rapidly develop and cope with changes; however, the main approach to improving the status of enterprises in the network is strengthening independent research and development by improving their technical level. Relevant research was consistent with the findings of Kong [37].
2. The relationship between the network scale and the knowledge transfer performance in the AI innovation network. It can be seen from the analysis results of the classified regression that the regression coefficient between the network scale and knowledge transfer performance was 0.256 (P<0.000), which indicates the scale of the innovation network has a certain positive effect on the network subject obtaining knowledge. Specifically, the larger the scale of the AI industry innovation network, the more network subjects are included in the whole network. Many network subjects possess a large amount of R & D knowledge, thus forming a network knowledge sharing platform. Based on the principle of exchange (exchanging what one has for what one needs), the probability of knowledge transfer between network subjects is greater, the content that knowledge recipients acquire is more abundant, and the transferred knowledge performance is better.
3. The relationship between the network relationship strength and knowledge transfer performance in the AI innovation network. The regression coefficient between network relationship strength and knowledge transfer performance was 0.065 (P < 0.245). This indicates that there was no significant positive correlation between these two variables. Uzzi [37] indicated that strong network relationships may produce “excessive embeddedness”, and if a firm is always in an innovation network with strong network relationship, it will create new obstacles to firm knowledge transfer and technological innovation rather than promote innovation, thus missing the opportunity to develop key technologies. The results of this paper also showed that strong network relationships are not conducive to knowledge transfer among enterprises but will lead them to fall into the established innovation network, making them unable to receive knowledge and technology from other channels, which is consistent with Uzzi’s research conclusions. The reason behind this result is that the strong relationship network formed in the innovation network tends to restrict the enterprise to the inherent relationship and is not conducive to the establishment of new relationships between the enterprise and other network entities in the innovation network.
4. The relationship between network stability and knowledge transfer performance in the AI innovation network. The regression coefficient between network relationship stability and knowledge transfer performance was 0.011 (P < 0.002) and failed to pass the test in the stepwise regression, which shows that the network relationship stability has no significant effect on the performance of knowledge transfer. The reason behind these results is that some network entities in the innovation network have formed trust in each other due to long-term technology or trade cooperation, thus evolving into a fixed network group. The entry of other enterprises will thus face certain restrictions, and the business relationship within the network remains stable. However, this kind of stable relationship is very unfavorable for the network in terms of receiving advanced knowledge resources and is also not conducive to enterprises learning new knowledge, thus hindering the development of innovation activities and knowledge transfer activities and making the performance of knowledge transfer low.
5. The relationship between reciprocity and knowledge transfer performance in the AI innovation network. The regression coefficient between network reciprocity and knowledge transfer performance was 0.022 (P < 0.152), which shows that there was no significant relationship between network reciprocity and knowledge transfer performance. In the process of innovation network operation, although the mutual benefit between enterprises has a certain effect on the trade relationship and distribution of interests, it has no obvious positive effect on knowledge transfer.

### Discussion of the regression results on organizational distance as independent variables

Through classified and stepwise regressions, we posit that was is a significant negative correlation between organizational cultural distance and knowledge transfer performance (the classified regression coefficient was -0.329, p < 0.000), and there was no significant correlation between geographic distance and the knowledge transfer performance among organizations. Nowadays, with the development of communication technology, geographical distance cannot be a factor hindering knowledge sharing and knowledge transfer among AI enterprises around the world. However, on a global scale, the organizational culture distance formed by different international enterprises due to different cultural backgrounds and different languages potentially hinders knowledge transfer performance. Therefore, if Chinese AI enterprises aim for integration into the innovation network, they should reduce differences in language and culture during the process of learning and practice exchange with foreign excellent enterprise employees. Specifically, the enterprise selects qualified technical personnel to send to the partners to study. In addition to having high technical ability, the resident personnel must also be proficient in foreign languages, so that all kinds of knowledge can be effectively transmitted back to the home-base Chinese enterprise. In terms of reducing cultural differences, China can make full use of the multi-level and multi-forms of cultural exchange by constructing international cultural exchange communication of a friendly and communicative nature, which can also eliminate prejudice and misconceptions about cultural differences among international technicians and guide them to establish more optimistic and fair cultural concepts and respect cultural differences. Moreover, knowledge transfer activities are effectively carried out via the active participation of technical talents from various countries during exchanges of world culture.

### Discussion of the regression results for transferred knowledge characteristics as independent variables

The classification regression results in this section show that knowledge implicitness was significantly negative related to knowledge transfer performance in the innovation network (the classified regression coefficient was -0.221, p < 0.000), while knowledge complexity and knowledge transfer performance among organizations were not significantly correlated with each other. Usually, in the event of implicit knowledge transfer, the transferor (we set the knowledge transfer provider as the foreign AI enterprise) to explain the knowledge is worse in the process of transferring knowledge. Hence, the knowledge receiver (we set the knowledge receiver as the domestic AI enterprise) does not easily understand this knowledge. Moreover, the knowledge receiver cannot apply the transferred knowledge to the research and development of his or her firm. Therefore, Chinese AI enterprises should pay attention to the ability to absorb and digest implicit knowledge when cultivating AI industry technicians. Besides, they should also make the tacit knowledge articulated and use the information technology to record the unencoded knowledge as much as possible, which aids the convenience of digestion. Additionally, our research results also show that the complexity of knowledge has no obvious correlation with the performance of organizational knowledge transfer. This indicates that, in today's highly developed information technology, knowledge complexity does not impede the demand of knowledge receivers for knowledge learning.

### Discussion of the regression results for knowledge receiver factors as independent variables

According to the classified regression, there was a significant positive correlation between knowledge receiver willingness and knowledge absorptive capacity and knowledge transfer performance (the classified regression coefficients respectively were 0.215, p < 0.000 and 0.458, p < 0.000). However, in the stepwise regression, these two factors had no significant effect on knowledge transfer performance. This shows that both factors affect the performance of knowledge transfer to a certain extent, but this effect is less than those of other factors. Therefore, in order to improve their technology level, Chinese AI enterprises should strengthen their willingness to accept knowledge and capacity building to absorb new knowledge for their grasp of international frontier knowledge.

## Conclusion

In the context of the global economic downturn, a new round of scientific and technological revolution, industrial transformation, and social progress driven by AI has been emerging, which has rekindled hope for the future development of the world economy. The integration of AI and other industries has been increasingly affecting individuals’ production and lifestyles in addition to the effective application of AI technology in logistics, manufacturing, medical treatment, agriculture, meteorology and other fields to help achieve sustainable development goals. The continuous development of AI technology and its application has become inseparable from the exchange and cooperation between global AI enterprises. The knowledge transfer occurring in the AI industry innovation network can not only promote the experience if sharing and technology exchange among all participants but also help all parties meet common challenges, avoid potential risks, and solve practical problems.

This paper empirically analyzed the influencing factors of knowledge transfer performance on Chinese enterprises in the global AI innovation network and proposed key factors that represent the characteristics of Chinese enterprises' development and affect improvements in knowledge transfer performance. Overall, we argue that there is still a gap between China and other developed countries in terms of the development of the AI industry, and China is committed to reducing this gap via knowledge learning in relevant technical fields. Knowledge transfer in these fields is less affected by the factors mentioned above; however, China has stepped into the forefront of certain technological fields and plays a key role in the innovation network, which has a great impact on the knowledge transfer performance of the AI industry innovation network on a whole. In the long-term, China's AI industry development should adhere to more open, diversified, and shared concepts to ensure thorough integration into the global AI industry innovation network. While realizing its own sustainable development, China should also work with other partners to achieve the relevant goals of the 2030 Agenda for Sustainable Development with the precise application of AI technologies.

Lastly, there are some limitations of our research. Specifically, data on the innovation network stems from the high-tech industry. therefore, its conclusions are not completely generalizable to other sectors. In future research, data from other industries should be included in analysis.
