# Supplementary material for: The impact of knowledge transfer performance on the artificial intelligence industry innovation network: An empirical study of Chinese firms
Source: PLoS One. 2020 May 18;15(5):e0232658. doi: 10.1371/journal.pone.0232658 (PMC7233593; doi:10.1371/journal.pone.0232658)
Supplement: Attachment — Submitted filename: Reviewer Comments for Manuscript.docx [file pone.0232658.s001.docx]

The results are interesting, and the influencing factors of knowledge transfer are analyzed in detail. However, this manuscript does not show the innovation points in detail, many of these views on Knowledge Transfer Performance are based on previous literature researches.

These are not some control variables that included in all the regression equations, it may overestimate the effect of knowledge transfer performance.

There should be a paragraph on the limitations of the study.

Lines 75-77: This postulate is not suit for all of Chinese AI enterprises. Currently, there are also many internationally influential and innovative enterprises in China. In addition, these measurement items only focus on other enterprises instead of foreign AI enterprises, the research postulate conflicts with measurement items.

Lines 86: ……”thus realizes”……or “thus realizing”

Lines 340-341: “while relationship strength, stability, and reciprocity were negatively related to knowledge transfer performance”, however the estimated coefficient in table 12 shows positively related to knowledge transfer performance.

Lines 413-414: how to get these conclusions, from Empirical test or literature, further evidence is needed to support these conclusions. This paragraph mainly discusses the relationship between the centrality characteristics of the enterprise and the knowledge transfer performance.
