# Supplementary material for: The impact of knowledge transfer performance on the artificial intelligence industry innovation network: An empirical study of Chinese firms
Source: PLoS One. 2020 May 18;15(5):e0232658. doi: 10.1371/journal.pone.0232658 (PMC7233593; doi:10.1371/journal.pone.0232658)
Supplement: Peer review history

# Peer review: Decision Letter 0

**DOI:** 10.1371/journal.pone.0232658.r001

**Authors:** Bing Xue

29 Jan 2020

PONE-D-19-34736

The Impact of Knowledge Transfer Performance on the Artificial Intelligence Industry Innovation Network: An Empirical Study of Chinese firms

PLOS ONE

Dear Dr. Ma,

Thank you for submitting your manuscript to PLOS ONE. After careful consideration, we feel that it has merit but does not fully meet PLOS ONE’s publication criteria as it currently stands. Therefore, we invite you to submit a revised version of the manuscript that addresses the points raised during the review process.

We would appreciate receiving your revised manuscript by Mar 14 2020 11:59PM. When you are ready to submit your revision, log on to https://www.editorialmanager.com/pone/ and select the 'Submissions Needing Revision' folder to locate your manuscript file.

If you would like to make changes to your financial disclosure, please include your updated statement in your cover letter.

To enhance the reproducibility of your results, we recommend that if applicable you deposit your laboratory protocols in protocols.io, where a protocol can be assigned its own identifier (DOI) such that it can be cited independently in the future. For instructions see: http://journals.plos.org/plosone/s/submission-guidelines#loc-laboratory-protocols

Please include the following items when submitting your revised manuscript:

- A rebuttal letter that responds to each point raised by the academic editor and reviewer(s). This letter should be uploaded as separate file and labeled 'Response to Reviewers'.
- A marked-up copy of your manuscript that highlights changes made to the original version. This file should be uploaded as separate file and labeled 'Revised Manuscript with Track Changes'.
- An unmarked version of your revised paper without tracked changes. This file should be uploaded as separate file and labeled 'Manuscript'.

Please note while forming your response, if your article is accepted, you may have the opportunity to make the peer review history publicly available. The record will include editor decision letters (with reviews) and your responses to reviewer comments. If eligible, we will contact you to opt in or out.

We look forward to receiving your revised manuscript.

Kind regards,

Bing Xue, Ph.D.

Academic Editor

PLOS ONE

Journal Requirements:

1. When submitting your revision, we need you to address these additional requirements.

Please ensure that your manuscript meets PLOS ONE's style requirements, including those for file naming. The PLOS ONE style templates can be found at

http://www.journals.plos.org/plosone/s/file?id=wjVg/PLOSOne_formatting_sample_main_body.pdf and http://www.journals.plos.org/plosone/s/file?id=ba62/PLOSOne_formatting_sample_title_authors_affiliations.pdf

2. Please provide additional details regarding participant consent. In the ethics statement in the Methods and online submission information, please ensure that you have specified (1) whether consent was informed and (2) what type you obtained (for instance, written or verbal, and if verbal, how it was documented and witnessed). If your study included minors, state whether you obtained consent from parents or guardians. If the need for consent was waived by the ethics committee, please include this information.

[Note: HTML markup is below. Please do not edit.]

Reviewers' comments:

Reviewer's Responses to Questions

**Comments to the Author**

1. Is the manuscript technically sound, and do the data support the conclusions?

The manuscript must describe a technically sound piece of scientific research with data that supports the conclusions. Experiments must have been conducted rigorously, with appropriate controls, replication, and sample sizes. The conclusions must be drawn appropriately based on the data presented. 

Reviewer #1: Partly

Reviewer #2: Partly

Reviewer #3: Partly

Reviewer #4: Yes

Reviewer #5: No

Reviewer #6: Partly

**********

2. Has the statistical analysis been performed appropriately and rigorously? 

Reviewer #1: Yes

Reviewer #2: Yes

Reviewer #3: No

Reviewer #4: Yes

Reviewer #5: Yes

Reviewer #6: Yes

**********

3. Have the authors made all data underlying the findings in their manuscript fully available?

The PLOS Data policy requires authors to make all data underlying the findings described in their manuscript fully available without restriction, with rare exception (please refer to the Data Availability Statement in the manuscript PDF file). The data should be provided as part of the manuscript or its supporting information, or deposited to a public repository. For example, in addition to summary statistics, the data points behind means, medians and variance measures should be available. If there are restrictions on publicly sharing data—e.g. participant privacy or use of data from a third party—those must be specified.

Reviewer #1: Yes

Reviewer #2: No

Reviewer #3: No

Reviewer #4: Yes

Reviewer #5: No

Reviewer #6: Yes

**********

4. Is the manuscript presented in an intelligible fashion and written in standard English?

PLOS ONE does not copyedit accepted manuscripts, so the language in submitted articles must be clear, correct, and unambiguous. Any typographical or grammatical errors should be corrected at revision, so please note any specific errors here.

Reviewer #1: Yes

Reviewer #2: Yes

Reviewer #3: No

Reviewer #4: No

Reviewer #5: Yes

Reviewer #6: Yes

**********

5. Review Comments to the Author

Please use the space provided to explain your answers to the questions above. You may also include additional comments for the author, including concerns about dual publication, research ethics, or publication ethics. (Please upload your review as an attachment if it exceeds 20,000 characters)

Reviewer #1: The results are interesting, and the influencing factors of knowledge transfer are analyzed in detail. However, this manuscript does not show the innovation points in detail, many of these views on Knowledge Transfer Performance are based on previous literature researches.

These are not some control variables that included in all the regression equations, it may overestimate the effect of knowledge transfer performance.

There should be a paragraph on the limitations of the study.

Lines 75-77: This postulate is not suit for all of Chinese AI enterprises. Currently, there are also many internationally influential and innovative enterprises in China. In addition, these measurement items only focus on other enterprises instead of foreign AI enterprises, the research postulate conflicts with measurement items.

Lines 86: ……”thus realizes”……or “thus realizing”

Lines 340-341: “while relationship strength, stability, and reciprocity were negatively related to knowledge transfer performance”, however the estimated coefficient in table 12 shows positively related to knowledge transfer performance.

Lines 413-414: how to get these conclusions, from Empirical test or literature, further evidence is needed to support these conclusions. This paragraph mainly discusses the relationship between the centrality characteristics of the enterprise and the knowledge transfer performance.

Reviewer #2: This research article illustrates the impact of knowledge transfer performance on the artificial industry innovation network which based on Chinese firms’ data properly. There are several problems in this article. First, there is little information about how these firms were chosen. There are a lot of companies in China, but the characteristics of these companies are not discussed (eg. The company size, the location, main business and so on). Does the chosen firms representative? Second, how this questionnaire constructed and what information was included. Does this questionnaire have reliability? Who answered the questionnaire? These entire questions should be discussed. Third, both theoretical and empirical evidence should be added. In the end, the discussion should link the prior hypothesis properly.

Reviewer #3: Thanks for the very interesting paper. The aim of the paper is very interesting but the content has some weaknesses in the present version, especially related to the motivation, methodology and the conclusions. Please find below my comments in detail.

General comments

- the authors did not state the contribution of the paper

- the paper does not provide descriptive analysis on the sample of data used

- the manuscript did not provide a discussion on the results compared with the literature.

- the conceptual model was not contrasted with the literature

- the paper does not provide a description on the Artificial Intelligence industry in china

Technical comments

- the paper does not provide a section on the methodologies used

- the factorial analysis was not evaluated with goodness of fit measures

Reviewer #4: Thank you for inviting me to review this manuscript. This appears to be an interesting study. The manuscript is well structured and written in a good manner. The authors have conducted a thorough literature review, and analysed information accurately and sufficiently.

The paper can be accepted with minor grammatical corrections. It is recommended that a native English speaker may conduct minor revisions.

It was a pleasure reading this manuscript. I wish the authors all the best.

Reviewer #5: the abstract and conclusion in the article is presented in generalized form, which can be written on general knowledge based. there is nothing in the article to verify the data which has been used. data analysis section is good but data authenticity is questionable. results discussion is also not up to the standers.

Reviewer #6: Dear Editor, thank you for giving me opportunity to read and assess the manuscript titled “The Impact of Knowledge Transfer Performance on the Artificial Intelligence Industry Innovation Network: An Empirical Study of Chinese firms”. I think it is a good piece of writing however there are following changes required before publications.

1. The introduction section lacks in problem statement and motivation of the study. There is immense need to give arguments in favor of conducting this study.

2. In the conceptual model and hypothesis, it is highly recommended to support the arguments with the support of theory.

3. The choice of conducting this study on Chinese firms should be justified in methodology part.

4. In the discussion section each result should be support by possible interpretations.

5. The policy implications need attention. There is an ample room to suggest more policy implications in the conclusion section.

6. At end, it is important to highlight the limitations of the study and recommend future directions.

**********

6. PLOS authors have the option to publish the peer review history of their article (what does this mean?). If published, this will include your full peer review and any attached files.

If you choose “no”, your identity will remain anonymous but your review may still be made public.

**Do you want your identity to be public for this peer review?** For information about this choice, including consent withdrawal, please see our Privacy Policy.

Reviewer #1: No

Reviewer #2: No

Reviewer #3: No

Reviewer #4: Yes: Kashifa Akbar

Reviewer #5: No

Reviewer #6: No

[NOTE: If reviewer comments were submitted as an attachment file, they will be attached to this email and accessible via the submission site. Please log into your account, locate the manuscript record, and check for the action link "View Attachments". If this link does not appear, there are no attachment files to be viewed.]

While revising your submission, please upload your figure files to the Preflight Analysis and Conversion Engine (PACE) digital diagnostic tool, https://pacev2.apexcovantage.com/. PACE helps ensure that figures meet PLOS requirements. To use PACE, you must first register as a user. Registration is free. Then, login and navigate to the UPLOAD tab, where you will find detailed instructions on how to use the tool. If you encounter any issues or have any questions when using PACE, please email us at figures@plos.org. Please note that Supporting Information files do not need this step.

Attachment Submitted filename: Reviewer Comments for Manuscript.docx Click here for additional data file.

# Author comment: Author response to Decision Letter 0

**DOI:** 10.1371/journal.pone.0232658.r002

21 Mar 2020

Dear Dr. Xue and reviewers,

Firstly, we thank you for your hard working, and we thank the anonymous reviewers for their valuable comments. At present, the 2019-nCoV is rampant, we believe that it is not only the warm flowers that come on schedule, but also everyone who is healthy after the epidemic. The main corrections in the paper and the response to the reviewers’ comments are as follows:

Reviewer #1:

1.Response to comment: (The results are interesting, and the influencing factors of knowledge transfer are analyzed in detail. However, this manuscript does not show the innovation points in detail, many of these views on Knowledge Transfer Performance are based on previous literature researches)

Response: We are very sorry for our negligence of not mentioning the contribution and originality of the paper. We have made correction according to the Reviewer’s suggestions as follows.

Therefore, it is valuable to explore the operation mode of the AI industry innovation network and identify the factors affecting the operation process. There is much existing research on the mechanism behind innovation network operation and knowledge transfer. The AI industry is an emerging industry and an important force in this new era of technological revolution; however, there are still only a few studies on the operation mechanism involved in the AI industry innovation network.

2.Response to comment: (These are not some control variables that included in all the regression equations, it may overestimate the effect of knowledge transfer performance.)

Response: In this paper, the independent variables used in the model settings are not controlled variables. Independent variables include Network centrality, Network scale, Relationship strength, Relationship stability, Reciprocity, Cultural distance, Geographic distance, Knowledge implicitness, Knowledge complexity, Willingness to receive, Knowledge absorptive capacity.

3.Response to comment: (There should be a paragraph on the limitations of the study.)

Response: It is really important and true that we need to highlight the limitations of the study and recommend future directions. We have modified as follows:

Lastly, there are some limitations of our research. Specifically, data on the innovation network stems from the high-tech industry. therefore, its conclusions are not completely generalizable to other sectors. In future research, data from other industries should be included in analysis.

4.Response to comment: (Lines 75-77: This postulate is not suit for all of Chinese AI enterprises. Currently, there are also many internationally influential and innovative enterprises in China. In addition, these measurement items only focus on other enterprises instead of foreign AI enterprises, the research postulate conflicts with measurement items.)

Response: As you said, Chinese AI companies do have some influence in the world. However, according to the survey, most of the AI companies in China are still relatively backward. Therefore, this article is based on the research hypothesis set in most cases in China. Besides, the measurement item is not only a measurement of Chinese enterprises, but also may include members of the innovation network. Meanwhile, Lines 307-319, we further explained the research objects, data measurement, etc.

5. Response to comment: (Lines 86: ……”thus realizes”……or “thus realizing”)

Response: In our revised manuscript, we have made correction as follows. “Thus realizes” has been changed into “thus realizing”.

6. Response to comment: (Lines 340-341: “while relationship strength, stability, and reciprocity were negatively related to knowledge transfer performance”, however the estimated coefficient in table 12 shows positively related to knowledge transfer performance.)

Response: We are very sorry that we have forgotten to fill in the coefficient symbols in the table due to mistakes. It is now more like the estimation coefficient in Table 12 below has been changed to negative correlation.

7. Response to comment: (Lines 413-414: how to get these conclusions, from Empirical test or literature, further evidence is needed to support these conclusions. This paragraph mainly discusses the relationship between the centrality characteristics of the enterprise and the knowledge transfer performance.)

Response: We have modified as follows: As you said, this paragraph explores the relationship between network characteristics and knowledge transfer performance. Relevant research is consistent with the findings of Kong (2017).

Reviewer #2:

1.Response to comment: (First, there is little information about how these firms were chosen. There are a lot of companies in China, but the characteristics of these companies are not discussed (eg. The company size, the location, main business and so on).)

Response: We are very sorry for our negligence of not discussing the characteristics of the companies. We have made correction according to the Reviewer’s suggestions as follows.

We first defined the AI companies participating in the innovation network. The definition criteria were based on the introduction on each company's official website and includes international AI technology research and development talents; Sino-foreign joint venture AI enterprises; and foreign-funded AI enterprises.

2.Response to comment: (Second, how this questionnaire constructed and what information was included. Does this questionnaire have reliability? Who answered the questionnaire? These entire questions should be discussed.)

Response: It is really true as reviewers suggested that we revised as follows.

The measurement items in this paper were closely centered around the research hypothesis of this study to ensure the content of the questionnaire was consistent with the survey items. At the same time, the design of the questionnaire items account for clarity, conciseness, and logic. Based on the maturity scale in the field of innovation networks, we drew on existing measurement items at home and abroad, and selects those that are widely used, highly recognized, and highly relevant to the purpose of this study. The questionnaire designed in this article consists of three parts: the first part is the introduction; the second part is the basic information of the investigator and the basic information of the unit; the third part is the main part of the questionnaire. The main body of the questionnaire uses Likert scale 5.

3.Response to comment: (Third, both theoretical and empirical evidence should be added. In the end, the discussion should link the prior hypothesis properly.)

Response: Theories and empirical descriptions are available, see text. The discussion is divided into five parts, which correspond to the five research hypotheses in the previous article. (1) The relationship between the network centrality and knowledge transfer performance in the AI innovation network. (2) The relationship between the network scale and the knowledge transfer performance in the AI innovation network. (3) The relationship between the network relationship strength and knowledge transfer performance in the AI innovation network. (4) The relationship between network stability and knowledge transfer performance in the AI innovation network. (5) The relationship between reciprocity and knowledge transfer performance in the AI innovation network.

Reviewer #3:

1.Response to comment: (the authors did not state the contribution of the paper)

Response: We are very sorry for our negligence of not mentioning the contribution and originality of the paper. We have made correction according to the Reviewer’s suggestions as follows.

Therefore, it is valuable to explore the operation mode of the AI industry innovation network and identify the factors affecting the operation process. There is much existing research on the mechanism behind innovation network operation and knowledge transfer. The AI industry is an emerging industry and an important force in this new era of technological revolution; however, there are still only a few studies on the operation mechanism involved in the AI industry innovation network.

2.Response to comment: (the paper does not provide descriptive analysis on the sample of data used)

Response: We are very sorry for our negligence of descriptive analysis on the sample of data of the paper. We have made correction according to the Reviewer’s suggestions as follows.

The descriptive analysis of the data sample is as follows. The proportion of companies in the startup stage was 2.75%; the proportion of companies in the investment stage was 11.95%; the proportion of companies in the growth stage was 69.79%; the proportion of companies in the mature stage was 14.64%; and the proportion of companies in the decline stage was 0.86%.

3.Response to comment: (the manuscript did not provide a discussion on the results compared with the literature.)

Response: The paper discusses the following three relationships in the discussion section and compares them with existing research literature. Discussion of the regression results of the innovation network characteristics as independent variables. Discussion of the regression results on organizational distance as independent variables. Discussion of the regression results for transferred knowledge characteristics as independent variables. 

4.Response to comment: (the conceptual model was not contrasted with the literature)

Response: The conceptual model in this paper is based on the existing research literature and the characteristics of the artificial intelligence industry.

5. Response to comment: (the paper does not provide a description on the Artificial Intelligence industry in China)

Response: It is really important and true that we need to provide a description on the Artificial Intelligence industry in china. In our revised manuscript, we have made correction as follows.

Since 2015, China has promulgated important national-level strategic plans, such as "Made in China 2025", "Guiding the Opinions of the State Council on Actively Implementing Internet Plus Actions", and the "New Generation AI Development Plan". Local governments have also actively issued policies supporting the development of the AI industry, which has led to an upsurge of AI development in China. At present, the development of Chinese AI is comprised of certain technical and industrial foundations. Specifically, a group of AI companies have gathered from the fields of chips, data, platforms, applications, among others, and have achieved staged results in some areas, developing them toward marketization. For example, AI has been applied in industries such as finance, security, and customer service. In some applications, the accuracy and efficiency of semantic, speech, face, and image recognition technologies have far surpassed manual. At this stage, a number of AI technologies in China, such as speech recognition, visual recognition, machine translation, and Chinese information processing, have reached international levels, forming a relatively complete industrial structure. However, when compared with developed countries, China still exhibits certain gaps in precision parts, the science and technology industry, industrial design, large-scale intelligent systems, and other fields.

6. Response to comment: (the paper does not provide a section on the methodologies used)

Response: This article uses questionnaires and uses stepwise regression for analysis. The basic idea of stepwise regression is to introduce the variables one by one into the model. After each explanatory variable is introduced, an F-test is performed, and the selected explanatory variables are subjected to a t-test one by one. When it is no longer significant, delete it. This ensures that only significant variables are included in the regression equation before each new variable is introduced. This is an iterative process until neither significant explanatory variable are selected into the regression equation, nor are insignificant explanatory variables removed from the regression equation. To ensure that the final set of explanatory variables is optimal.

7. Response to comment: (the factorial analysis was not evaluated with goodness of fit measures)

Response: We have modified as follows:

We also analyzed the nominal variables by utilizing Eigenvalues, Cumulative interpretation variation, Factor load, Cronbach’s α and other mensuration as follows.

Reviewer #4:

1.Response to comment: (The paper can be accepted with minor grammatical corrections. It is recommended that a native English speaker may conduct minor revisions.)

Response: It is really true as reviewers suggested that we need to modify the grammar of the article.

We have rechecked the language of this study.

Reviewer #5:

1.Response to comment: (the abstract and conclusion in the article is presented in generalized form, which can be written on general knowledge based. there is nothing in the article to verify the data which has been used. data analysis section is good but data authenticity is questionable. results discussion is also not up to the standers.)

Response: 

The abstract and conclusions of this article have been modified based on editorial comments. The data source is modified as follows. Results discussion was analyzed based on the results of the questionnaire.

We first defined the AI companies participating in the innovation network. The definition criteria were based on the introduction on each company's official website and includes international AI technology research and development talents; Sino-foreign joint venture AI enterprises; and foreign-funded AI enterprises. In order to obtain a sufficient sample of effective questionnaires, we defined companies that meet any of the above conditions as AI companies integrated into the innovation network. The selection of this questionnaire was mainly aimed at AI companies participating in technology R&D personnel or middle and senior managers of the enterprise, to ensure the questionnaire participants’ comprehensive understanding of each company's participation in the AI industry innovation network.

Reviewer #6:

1.Response to comment: (The introduction section lacks in problem statement and motivation of the study. There is immense need to give arguments in favor of conducting this study.)

Response: We are very sorry for our negligence of not mentioning the contribution and originality of the paper. We have made correction according to the Reviewer’s suggestions as follows,

Therefore, it is valuable to explore the operation mode of the AI industry innovation network and identify the factors affecting the operation process. There is much existing research on the mechanism behind innovation network operation and knowledge transfer. The AI industry is an emerging industry and an important force in this new era of technological revolution; however, there are still only a few studies on the operation mechanism involved in the AI industry innovation network.

2.Response to comment: (In the conceptual model and hypothesis, it is highly recommended to support the arguments with the support of theory.)

Response: The research hypotheses and research frameworks proposed in this article are documentable

3.Response to comment: (The choice of conducting this study on Chinese firms should be justified in methodology part.)

Response: we revised as Lines 307-309: We first defined the AI companies participating in the innovation network. The definition criteria were based on the introduction on each company's official website and includes international AI technology research and development talents, Sino-foreign joint venture AI enterprises, and foreign-funded AI enterprises.

4.Response to comment: (In the discussion section each result should be support by possible interpretations.)

The paper discusses the following three relationships in the discussion section and compares them with existing research literature. Discussion of the regression results of the innovation network characteristics as independent variables. Discussion of the regression results on organizational distance as independent variables. Discussion of the regression results for transferred knowledge characteristics as independent variables.

5. Response to comment: (The policy implications need attention. There is an ample room to suggest more policy implications in the conclusion section.)

Response: It is really important and true that we need to explain the policy implications. In our revised manuscript, we have made correction as follows.

Overall, we argue that there is still a gap between China and other developed countries in terms of the development of the AI industry, and China is committed to reducing this gap via knowledge learning in relevant technical fields. Knowledge transfer in these fields is less affected by the factors mentioned above; however, China has stepped into the forefront of certain technological fields and plays a key role in the innovation network, which has a great impact on the knowledge transfer performance of the AI industry innovation network on a whole. In the long-term, China's AI industry development should adhere to more open, diversified, and shared concepts to ensure thorough integration into the global AI industry innovation network. While realizing its own sustainable development, China should also work with other partners to achieve the relevant goals of the 2030 Agenda for Sustainable Development with the precise application of AI technologies.

6. Response to comment: (At end, it is important to highlight the limitations of the study and recommend future directions.)

Response: It is really important and true that we need to highlight the limitations of the study and recommend future directions. We have modified as follows:

Lastly, there are some limitations of our research. Specifically, data on the innovation network stems from the high-tech industry. therefore, its conclusions are not completely generalizable to other sectors. In future research, data from other industries should be included in analysis.

Attachment Submitted filename: Response to Reviewers.docx Click here for additional data file.

# Peer review: Decision Letter 1

**DOI:** 10.1371/journal.pone.0232658.r003

**Authors:** Bing Xue

21 Apr 2020

The Impact of Knowledge Transfer Performance on the Artificial Intelligence Industry Innovation Network: An Empirical Study of Chinese firms

PONE-D-19-34736R1

Dear Dr. Ma,

We are pleased to inform you that your manuscript has been judged scientifically suitable for publication and will be formally accepted for publication once it complies with all outstanding technical requirements.

Within one week, you will receive an e-mail containing information on the amendments required prior to publication. When all required modifications have been addressed, you will receive a formal acceptance letter and your manuscript will proceed to our production department and be scheduled for publication.

Shortly after the formal acceptance letter is sent, an invoice for payment will follow. To ensure an efficient production and billing process, please log into Editorial Manager at https://www.editorialmanager.com/pone/, click the "Update My Information" link at the top of the page, and update your user information. If you have any billing related questions, please contact our Author Billing department directly at authorbilling@plos.org.

If your institution or institutions have a press office, please notify them about your upcoming paper to enable them to help maximize its impact. If they will be preparing press materials for this manuscript, you must inform our press team as soon as possible and no later than 48 hours after receiving the formal acceptance. Your manuscript will remain under strict press embargo until 2 pm Eastern Time on the date of publication. For more information, please contact onepress@plos.org.

With kind regards,

Bing Xue, Ph.D.

Academic Editor

PLOS ONE

Additional Editor Comments (optional):

Reviewers' comments:

Reviewer's Responses to Questions

**Comments to the Author**

1. If the authors have adequately addressed your comments raised in a previous round of review and you feel that this manuscript is now acceptable for publication, you may indicate that here to bypass the “Comments to the Author” section, enter your conflict of interest statement in the “Confidential to Editor” section, and submit your "Accept" recommendation.

Reviewer #1: All comments have been addressed

Reviewer #2: All comments have been addressed

Reviewer #3: All comments have been addressed

Reviewer #4: All comments have been addressed

Reviewer #6: All comments have been addressed

**********

2. Is the manuscript technically sound, and do the data support the conclusions?

The manuscript must describe a technically sound piece of scientific research with data that supports the conclusions. Experiments must have been conducted rigorously, with appropriate controls, replication, and sample sizes. The conclusions must be drawn appropriately based on the data presented. 

Reviewer #1: Yes

Reviewer #2: Yes

Reviewer #3: Yes

Reviewer #4: Yes

Reviewer #6: Yes

**********

3. Has the statistical analysis been performed appropriately and rigorously? 

Reviewer #1: Yes

Reviewer #2: Yes

Reviewer #3: Yes

Reviewer #4: Yes

Reviewer #6: Yes

**********

4. Have the authors made all data underlying the findings in their manuscript fully available?

The PLOS Data policy requires authors to make all data underlying the findings described in their manuscript fully available without restriction, with rare exception (please refer to the Data Availability Statement in the manuscript PDF file). The data should be provided as part of the manuscript or its supporting information, or deposited to a public repository. For example, in addition to summary statistics, the data points behind means, medians and variance measures should be available. If there are restrictions on publicly sharing data—e.g. participant privacy or use of data from a third party—those must be specified.

Reviewer #1: Yes

Reviewer #2: (No Response)

Reviewer #3: Yes

Reviewer #4: Yes

Reviewer #6: Yes

**********

5. Is the manuscript presented in an intelligible fashion and written in standard English?

PLOS ONE does not copyedit accepted manuscripts, so the language in submitted articles must be clear, correct, and unambiguous. Any typographical or grammatical errors should be corrected at revision, so please note any specific errors here.

Reviewer #1: Yes

Reviewer #2: Yes

Reviewer #3: Yes

Reviewer #4: Yes

Reviewer #6: Yes

**********

6. Review Comments to the Author

Please use the space provided to explain your answers to the questions above. You may also include additional comments for the author, including concerns about dual publication, research ethics, or publication ethics. (Please upload your review as an attachment if it exceeds 20,000 characters)

Reviewer #1: I really appreciate this work. From my end there seems to be either any major nor any minor comments. Good job

Reviewer #2: The author of this paper addressed all the comments properly. The reviewer think this paper may published in this journal.

Reviewer #3: (No Response)

Reviewer #4: The author/s have responded to the concerns. The quality of the manuscript is improved. I wish the author/s good luck.

Reviewer #6: (No Response)

**********

7. PLOS authors have the option to publish the peer review history of their article (what does this mean?). If published, this will include your full peer review and any attached files.

If you choose “no”, your identity will remain anonymous but your review may still be made public.

**Do you want your identity to be public for this peer review?** For information about this choice, including consent withdrawal, please see our Privacy Policy.

Reviewer #1: Yes: Tao Bu, Beijing Jiaotong University

Reviewer #2: No

Reviewer #3: No

Reviewer #4: No

Reviewer #6: Yes: Muhammad Awais Baloch

# Editor report: Acceptance letter

**DOI:** 10.1371/journal.pone.0232658.r004

**Authors:** Bing Xue

6 May 2020

PONE-D-19-34736R1 

The Impact of Knowledge Transfer Performance on the Artificial Intelligence Industry Innovation Network: An Empirical Study of Chinese firms 

Dear Dr. Ma:

I am pleased to inform you that your manuscript has been deemed suitable for publication in PLOS ONE. Congratulations! Your manuscript is now with our production department. 

If your institution or institutions have a press office, please notify them about your upcoming paper at this point, to enable them to help maximize its impact. If they will be preparing press materials for this manuscript, please inform our press team within the next 48 hours. Your manuscript will remain under strict press embargo until 2 pm Eastern Time on the date of publication. For more information please contact onepress@plos.org.

For any other questions or concerns, please email plosone@plos.org. 

Thank you for submitting your work to PLOS ONE.

With kind regards,

PLOS ONE Editorial Office Staff

on behalf of

Professor Bing Xue 

Academic Editor

PLOS ONE
